# Supplementary material for: Regardless of the Brewing Conditions, Various Types of Tea are a Source of Acetylcholinesterase Inhibitors
Source: Nutrients. 2020 Mar 6;12(3):709. doi: 10.3390/nu12030709 (PMC7146204; doi:10.3390/nu12030709)
Supplement: Supplementary file 1 [file nutrients-12-00709-s001.pdf]

Table S1. Significance of differences in tested tea. AChE inhibition.

Tea samples	Brewing time (min)	Brewing temperatures					
		100°C	95°C	90°C	85°C	80°C	75°C
1	5						a
	10						
	15						b
2	5				b		
	10				b		
	15				a		
3	5						b
	10						
	15						a
4	5	a	a	b	b		
	10		b	b	a		
	15	b	b	a	b		
5	5	b			b	a	
	10	a			b	b	
	15	b			a		
6	5					b	b
	10					b	a
	15					a	b
7	5		b			b	
	10		a		a	a	
	15				b	b	
8	5						
	10						b
	15						a
9	5	a			b		
	10					a	
	15	b			a	b	
10	5						
	10						
	15						
11	5		b	b	b	b	
	10		b	a	a	a	
	15		a	b			
12	5					a	
	10					b	b
	15					b	a
13	5				b		
	10			a	a		
	15			b	b		
14	5		a	b	b	a	
	10		b	a		b	
	15			b	a	b	

Legends: 1- Biofix Tea Raspberry, 2- Biofix Tea Multi-fruit, 3- Biofix Tea Wild Strawberry, 4- Bio fix Tea Cranberry, 5- Biofix Tea Wild Rose, 6- Biofix Green Tea with Quince, 7- Biofix Green Tea Guarana and Passion, 8- Biofix Green Tea Ginseng and Pomegranate, 9- Biofix Green Tea Original, 10- Teekanne White Tea, 11- Irving White Tea Pomegranate and Gooseberries, 12- Tetley Black Tea, 13- Lipton Black Tea, 14- Saga Black Tea; Within each brew and a given brewing time, various small letters a, b… denote significant differences ($p < 0.05$) of brews produced at various brewing temperatures.

Table S2. Significance of differences in tested tea. AChE inhibition.

Brewing temperatures	Tea samples	Brewing time (min) 5	10	15	Tea samples	Brewing time (min) 5	10	15
100°C								
95°C			A					
90°C	1				8			
85°C			B					
80°C								
75°C								
100°C		B	A			A		
95°C		A, B	A, B					B
90°C	2	B, C			9	B		B
85°C		A, C	B, C			B		B
80°C		B, C				B		A
75°C		C	C					
100°C		C	A, C, D			A		
95°C		C	C, D					
90°C	3	C	C, D		10			
85°C		A, C	B, C					
80°C		B	A, B, D					
75°C		A, B	A, B			B		
100°C				B, C		A, B	A, B, D	
95°C				C		B	A-C	B
90°C	4			A, C	11	B	C, D	A
85°C				C		C	D	
80°C				C		C	B-D	B
75°C				A, B		A, C	A, D	
100°C		B, C	A			A		
95°C		B, C						
90°C	5	A, C	A, B		12		A	
85°C		A, C		B		B		
80°C		B, C	B, C				B	
75°C		A, B	C				B	
100°C		B	C, D	B				A
95°C		A	A, B, D	B				A
90°C	6		D	B	13			
85°C			A, D	B				
80°C		B	B-D	A				B
75°C		B	A-C					B
100°C		B		B		A	A-C	A
95°C		B	A	B		A	A, B, D	
90°C	7	A			14	B	B-D	B
85°C		B		A		B	C, D	B
80°C		A	B	B		B	A, D	B
75°C		B		B		B	D	

Legends: 1- Biofix Tea Raspberry, 2- Biofix Tea Multi-fruit, 3- Biofix Tea Wild Strawberry, 4- Bio fix Tea Cranberry, 5- Biofix Tea Wild Rose, 6- Biofix Green Tea with Quince, 7- Biofix Green Tea Guarana and Passion, 8- Biofix Green Tea Ginseng and Pomegranate, 9- Biofix Green Tea Original, 10- Teekanne White Tea, 11- Irving White Tea Pomegranate and Gooseberries, 12- Tetley Black Tea, 13- Lipton Black Tea, 14- Saga Black Tea; Within each brew and a given brewing temperature, various capital letters A, B…denote significant differences ($p < 0.05$) of brews produced at various brewing times.
